# Esthetic, mechanical, and biological outcomes of various implant abutments for single-tooth replacement in the anterior region: a systematic review of the literature

**DOI:** 10.1186/s40729-021-00370-7

**Published:** 2021-09-08

**Authors:** Dimitra Totou, Olga Naka, Shamir B. Mehta, Subir Banerji

**Affiliations:** 1 Faculty of Dentistry Oral and Craniofacial Sciences, King’s College London, London, UK; 2 School of Dentistry, Aristotle University of Thessaloniki, 54124 Thessaloniki, Greece

**Keywords:** Implant abutment, Zirconia abutment, Single-implant restoration, Esthetics, Anterior implants

## Abstract

**Background:**

The choice of the appropriate implant abutment is a critical step for a successful outcome. Titanium abutments have demonstrated high survival rates, due to their excellent biocompatibility and high mechanical strength, although they often result in a grayish discoloration of the peri-implant mucosa. This esthetic concern culminated in the introduction of ceramic abutments. The aim of this review was to assess the esthetic, mechanical, and biological outcomes as well as the survival of the different types of abutments used for single-implant restorations in the anterior area.

**Material and methods:**

An electronic search was conducted in Medline, Embase, and Cochrane Central databases using the appropriate Mesh terms and predetermined eligibility criteria. The quality of the studies was assessed using the ROB 2 tool. The last search was conducted on 18th of March 2020.

**Results:**

From the 2074 records initially identified, 23 randomized controlled trials (32 publications) were included for qualitative analysis. Data were classified based on study information, specific characteristics of the intervention and comparator, and information related to the outcome measures. Seven studies exhibited an overall low risk of bias, while twelve studies raised some concerns.

**Conclusions:**

The rate of abutment failure was low and was associated with the ceramic abutments, especially those with internal connection. Limited correlation was noted between soft tissue thickness and color difference. Titanium abutments caused significantly more discoloration to the soft tissues than ceramic abutments, while hueing (gold or pink) slightly improved their color performance. Zirconia allowed a better color match than titanium or gold abutments, still discolored slightly the soft tissues. The submucosally modified zirconia abutments exhibited encouraging results. No significant difference was reported between materials or different types of retention on recession, papillary fill, and biological outcomes.

## Background

Dental implant placement for a single anterior tooth rehabilitation is shown to have high survival rates [1]. The criteria for implant survival over time, include the biological integration of the implant, the absence of mechanical complications, and the esthetic integration of the restoration with the adjacent teeth [2, 3]. The latter parameter is multifactorial. The gingival margin levels, the interproximal papilla, the soft tissue contour, the color of the tissues, the color, anatomy, and texture of the restoration should mimic those of the adjacent natural teeth [4, 5].

The choice of the appropriate implant abutment is a critical step for a successful outcome. Titanium abutments have demonstrated high survival rates [6–8], due to their excellent biocompatibility and high mechanical strength [9]. Nevertheless, metallic abutments often result in a grayish discoloration of the peri-implant mucosa, especially in thin biotype soft tissues [10–13].

This esthetic concern culminated in the introduction of ceramic abutments. However, the early designs of ceramic abutment manufactured from densely sintered alumina were associated with an increased risk of fracture [14, 15]. Yttrium-stabilized zirconia for CAD-CAM abutments on the other hand have exhibited increased mechanical strength compared to alumina, and excellent biocompatibility comparable to that of titanium [16–19]. However, zirconia has been associated with lower fracture resistance than titanium [20, 21], as it is a more brittle material. Systematic reviews comparing ceramic with metal abutments have shown no significant difference in the technical complication rates or the survival rates [7, 8]. Notably, these systematic reviews assessed abutments in both anterior and posterior areas, supporting both single-implant restorations and fixed partial dentures, and included mainly abutments with an external connection. Evidence suggests that even zirconia abutments fail to completely integrate with the natural teeth due to their bright white color which can result in blanching of the peri-implant mucosa [22]. Therefore, a novel concept of modifying the zirconia abutments, either by using a fluorescent zirconia material or by veneering the submucosal part of the abutment with pink or fluorescent porcelain has been introduced [23, 24].

There is a plethora of current research on the esthetics of anterior single-implant restorations; however, the interpretation of the data can be confusing due to the heterogenicity of the studies. There are systematic reviews based exclusively on assessing the treatment outcomes of zirconia abutments, while the metallic abutments have not been addressed [25, 26]. Moreover, the review question of some systematic reviews focused not only on single implants in the anterior area but also in the posterior area [25, 27]. As the mechanical and esthetic requirements of implant restorations in the anterior and posterior regions are not identical, their combination can lead to erroneous interpretations of the results. Furthermore, the eligibility criteria of some reviews included evidence from case reports and case series to reach their conclusions, which are low in the hierarchy of evidence [28]. The most recent systematic review comparing the esthetic, mechanical, and biological outcomes of various implant abutment designs in the anterior region was conducted by Bidra et al. in 2013 [29]. However, in the last 7 years, new advances in implant dentistry have resulted in the introduction of new evidence as well as the introduction of contemporary abutment materials and abutment configurations.

Thus, the aim of this systematic review was to review the up to date evidence and assess the esthetic, biological, and mechanical outcomes alongside the survival of the different types of abutments used for single-implant restorations in the anterior region. The objectives of the study were to identify and assess the effect of each type of implant abutment on specific esthetic parameters relating to pink and white esthetics, biological parameters, mechanical complications, and the different modes of failure.

## Material and methods

This systematic review was conducted following the PRISMA statement for reporting systematic reviews [30]. A protocol for this review was agreed beforehand and was registered in PROSPERO (CRD42020204083).

The PICO (Population, Intervention, Comparison, Outcome) criteria framed the following research question: In adults restored with single-implant abutment in the upper and/or lower anterior zone (first bicuspid to first bicuspid), which implant abutment (zirconia; alumina; titanium; cast metal abutment) performs best in terms of esthetic, mechanical, biological and survival outcomes?

### Eligibility criteria

The studies included had to be randomized clinical trials, published in peer reviewed journals in English language. The full text of the studies had to be retrievable. Non-randomized clinical studies, observational clinical studies, case reports, case series, in vitro studies, and studies related to implant abutments for a provisional restoration were excluded.

### Search strategy

Studies were identified through electronic search of the Ovid Medline, Embase, and Cochrane Central Register of Controlled Trials (CENTRAL) databases. Moreover, the references of included articles were hand-searched for relevant studies. All databases were searched with no time filter. The following combination of keywords was used: (“single implant in the aesthetic zone” OR “single tooth replacement” OR “single dental implant” OR “single-implant reconstruction” OR “dental implants, single- tooth” [MeSh term] OR “single-implant crown” OR “single-tooth implant” OR “implant-supported crowns” OR “dental prosthesis, implant-supported” [MeSh term]) AND (“implant abutment” OR “zirconia abutment” OR zirconium [Mesh term] OR “titanium abutment” OR “alumina abutment” OR “ceramic abutment” OR “esthetic abutment” OR “CAD-CAM abutment” OR “dental abutments” [MeSh term] OR “custom abutment” OR “dental implant-abutment design” [MeSh term] OR “gold-alloy abutment” OR “titanium base” OR “individualized abutment” OR “alumina-toughened zirconia abutment”) AND (“survival rate” [MeSh term] OR “success rate” OR “survival analysis” [MeSh term] OR “mechanical outcomes” OR “technical outcomes” OR “biological outcomes” OR “clinical outcomes” OR “clinical performance” OR “esthetic outcomes” OR “esthetic performance” OR “esthetics, dental” [MeSh term] OR “technical complication” OR “biological complication” OR “retention” OR “screw loosening” OR “screw fracture” OR “fracture” OR “chipping” OR “spectrophotometric analysis” OR “discoloration” OR “color change” OR “peri-implant soft tissue” OR “peri-implant mucosa” OR “soft tissue reaction” OR “dental prosthesis repair” [MeSh term] OR “dental restoration failure” [MeSh term]).

The last database search was conducted on 18th of March 2020.

The study selection process was performed by two independent reviewers, reducing the possibility of rejecting relevant reports. Any disagreements were resolved debating with the other authors.

Data were classified based on study information, specific characteristics of the intervention and comparator, and information related to the outcome measures. Articles with the same sample were summarized as one study. In cases where the same research team had multiple publications of the same trial, the most recent one was included, unless a previous publication had different sample size. When several publications of the same project reported different outcomes, the relevant data were extracted and presented in the most recent publication.

The findings of the individual studies were combined to reveal similarities and differences across the primary studies. The methods used to generate the research question were grouped as well as the outcomes, where possible quantitative synthesis was performed. Where the included studies were heterogeneous mainly in terms of design and methodology, qualitative interpretation of the results was performed.

The quality of the included studies was assessed using the Cochrane Risk of Bias tool (ROB 2) [31].

## Results

### Study selection

**Fig. 1:**
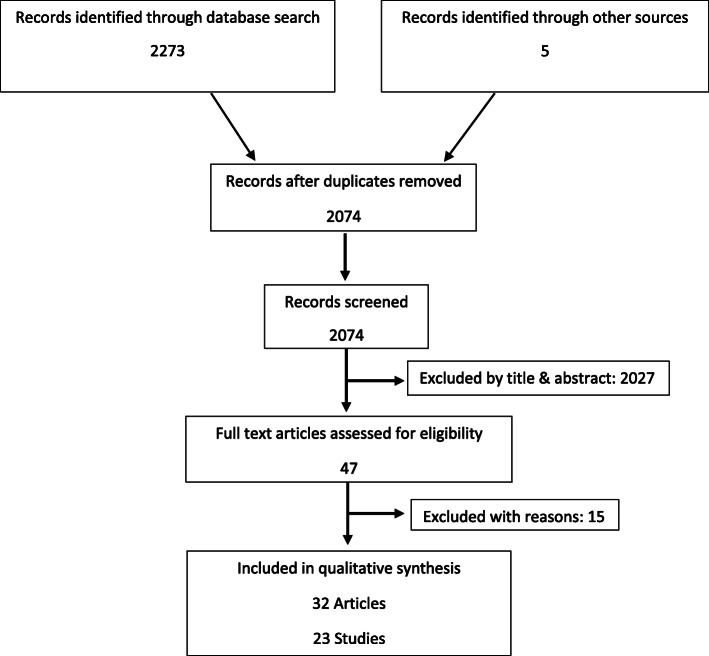
Flow diagram of study selection process

The study selection process is illustrated in Fig. 1. Forty-seven articles were assessed for eligibility through full-text reading while 15 of them were excluded with reasons, as described in Table 1 [32–46]. Moreover, 9 articles were publications of the same clinical trial, but had a different sample size or outcomes (Table 2). Therefore, 23 studies (32 publications) were included in this systematic review.

**Table 1:** Excluded articles with reasons

Excluded articles	Reason of exclusion
Zembic et al. 2009 [32], Sailer et al. 2009 [33], Zembic et al. 2013 [34], Bosch et al. 2018 [35]	Included molar teeth
Van Brakel et al. 2012 [36], den Hartog et al. 201 3[37], Rieder et al. 2016 [38]	Irrelevant outcomes
Furze et al. 2012 [39], Montero et al. 2012 [40], Schwarz et al. 2012 [41], Hosseini et al. 2013 [42], Fenner et al. 2016 [43], Kim et al. 2016 [44], Peng et al. 2017 [45], Asgeirsson et al. 2019 [46]	Study type (not RCTs)

**Table 2:** Multiple publications of the same study

• Büchi et al. 2014 [23], Thoma et al. 2016a [47] and Brandenberg et al. 2017 [48] (different outcomes) • Eisner et al. 2018 [49] is the 3-year follow-up of the abovementioned study (different sample due to loss of follow-up) • Laass et al. 2019 [50] is the 5-year follow-up of the abovementioned study (different sample due to loss of follow-up)	
• Gallucci et al. 2011a and Gallucci et al. 2011b [51, 52] (different outcomes)	
• Heierle et al. 2019 [53] is the 3 year follow-up of Thoma et al. 2018a [54] (different sample due to loss of follow-up)	
• Thoma et al. 2016b [55] and Thoma et al. 2018b (different outcomes) [56] • Kraus et al. 2019 [57] is the 3 year follow-up of Thoma et al. 2018b [56] (different sample size due to loss of follow-up)	
• Wittneben et al. 2017 [58] and Wittneben et al. 2020 [59] (same sample size)	
• Patil et al. 2014 [60] and Patil et al. 2016 [61] and Patil et al. 2017 [62] (different outcomes)	
• Gil et al. 2017 [63] and Gil et al. 2019 [64] and Bittner et al. 2020 [65] (different outcomes)	

All studies included abutments placed in the anterior maxilla and 9 of them reported implants placed in the anterior mandible (Table 3). Regarding the abutment material type, 7 studies reported outcomes of zirconia (Zr) abutments [50, 53, 57, 59, 69, 72, 73], 6 studies compared zirconia with titanium (Ti) and other metal abutments (2 Zr vs Ti [67, 68], 1 Zr vs gold alloy [66], 3 Zr vs pink Ti/Ti/gold Ti [22, 70, 71]), 3 studies compared alumina with metallic abutments (2 with Ti [13, 15], 1 with gold [51]) and 3 studies compared different titanium abutments (2 compared abutment macro-design [60, 74] and 1 abutment color; pink vs conventional gray [65]). Both prefabricated [15, 50, 51, 57–61, 73] and customized [12, 21, 22, 46–49, 52–56, 62–68, 70–72] abutments were used.

**Table 3:** Descriptive characteristics of RCTs

Study	No_ patients (baseline)	No_ abutments follow-up (baseline)	Abutment location	Follow-up (years)	Intervention and control abutment type
Andersson et al. 2001 [15]	75	85 (89)	Maxilla and mandible	1-3	Prefabricated alumina/prefabricated Ti
Jung et al. 2008 [13]	30	30	N/A	N/A	Customized Alumina based/customized Ti or gold
Bressan et al. 2011 [22]	20	20	Maxilla	N/A	Customized Ti, gold alloy and Zr/contralateral tooth
Galluci et al. 2011a [51] Galluci et al. 2011b [52]	20	17 (20)	Maxilla	2	Prefabricated Ti with In-Ceram alumina blank/prefabricated Ti with PFM (cast-on gold coping)
Hosseini et al. 2011 [66]	36	75	Maxilla and mandible	1	Prefabricated Zr/prefabricated metallic
Büchi et al. 2014, Thoma et al. 2016a, Brandenberg et al. 2017 [23, 47, 48]	20	20	Maxilla and mandible	1	Customized Zr with pink ceramic submucosal veneering/customized Zr without veneering
Carrillo de Albornoz et al. 2014 [67]	30	25 (30)	Maxilla	1	Prefabricated Zr/prefabricated Ti
Patil et al. 2014, Patil et al. 2016, Patil et al. 2017 [60–62]	26	52	Maxilla and mandible	1	Prefabricated curved grooved Ti/ Prefabricated conventional divergent Ti
Baldini et al. 2016 [68]	24	22 (24)	Maxilla and mandible	1	Prefabricated Zr/prefabricated Ti
Paolantoni et al. 2016 [69]	65	74	Maxilla	4	Customized Zr (1 piece)/customized Zr (2 pieces)
Thoma et al. 2016b [55], Thoma et al. 2018b [56]	44	43 (44)	Maxilla and mandible	1	Customized Zr (screwed)/customized Zr (cemented) with or without submucosal veneering
Gil et al. 2017, Gil et al. 2019, Bittner et al. 2020 [63–65]	40	33 (40)	Maxilla	6 months	Customized, pink-anodized Ti/customized gray Ti
Lops et al. 2017 [70]	15	15	Maxilla and mandible	N/A	Customized Ti, gold alloy and Zr/contralateral tooth
Martinez-Rus et al. 2017 [71]	20	20	Maxilla	N/A	Customized Ti, gold-anodized Ti, pink hued Ti, and Zr on Ti-base/contralateral tooth
Thoma et al. 2017 [72]	24	24	Maxilla and mandible	N/A	Customized fluorescent Zr on Ti-base/customized Zr
Wittneben et al. 2017 [58], Wittneben et al. 2020 [59]	40	39 (40)	Maxilla	3	Prefabricated Zr/CAD/CAM Zr
Amorfini et al. 2018 [73]	32	30 (32)	Maxilla	10	Customized Zr with ceramic veneer fused on abutment/customized Zr
Eisner et al. 2018 [49]	20	18 (20)	Maxilla and mandible	3	Customized Zr with pink ceramic submucosal veneering/customized Zr without veneering
Thoma et al. 2018a [54]	33	33	Maxilla and mandible	6 months	Customized Zr directly veneered with veneering ceramic/customized Zr
Heierle et al. 2019 [53]	27	27	Maxilla and mandible	3	Customized Zr directly veneered with veneering ceramic/customized Zr
Laass et al. 2019 [50]	20	16 (20)	Maxilla and mandible	5	Customized Zr with pink ceramic submucosal veneering/customized Zr
Kraus et al. 2019 [57]	44	32 (44)	Maxilla and mandible	3	Customized Zr (screwed)/customized Zr (cemented) with or without submucosal veneering
Koutouzis et al. 2019 [74]	28	26 (27)	Maxilla	1	Customized Ti convex/customized Ti concave profile

Most ceramic abutments were restored with all-ceramic cemented [13, 15, 22, 50, 53, 57, 65, 67, 69–71, 73] or screw retained [13, 15, 51, 53, 57, 59, 69, 72, 73] crowns. The metallic abutments were restored with either cemented [13, 15, 60, 66, 68, 74] or screw retained [13, 51] metal-ceramic (PFM) crowns.

#### Mechanical outcomes

Eleven studies (14 articles) explored the mechanical parameters of the abutments (Table 4). Only 6 of these studies reported zirconia or alumina abutment fracture [15, 50, 53, 57, 67, 73]. Nineteen abutments fractured from a total of 446 while 5 fractured at the time of placement during tightening or at the laboratory procedures [15]. Notably, no fractures were reported in the metallic abutments. The fracture rate varied between 3 and 14%. Most fractures were assessed in internally connected ceramic customized abutments. Screw loosening was considered a minor complication and was reported only in 2 studies of zirconia abutments at a rate of 6% [49, 73]. Complications of the crowns supported by the abutments were also reported. The minor chipping of the veneering ceramic was the most common complication [50, 51, 54, 57, 66, 73]. Only 4 studies reported major fracture of the ceramic (all-ceramic restorations) that required crown replacement [15, 53, 58, 69]. Loss of crown retention was also reported in 2 studies [66, 73]. The mechanical failure rate for each study was calculated as the number of mechanical failures divided by the number of implants and then multiplied by 100 (Table 4).

**Table 4:** Mechanical outcomes

Study	Criteria	Abutment fracture	Abutment loosening	Prosthesis complication	Abutment fracture rate	Mechanical failure rate
Andersson et al. 2001 [15]	N/A	5 during prep or placement 2 minor chip fractures (B) 2 after loading (1 M and 7 M)	No	crown fracture of c group at 2 Y	7%	8.99%
Galluci et al. 2011b [51]	N/A	No	No	2 minor ceramic chipping in t	No	10.00%
Hosseini et al. 2011 [66]	N/A	No	No	1 ceramic chipping and 1 crown loss of retention at 1Y in metal group	No	2.67%
Thoma et al. 2016a [47]	USPHS	1 in t before 1 Y	No	1 chipping (in t) and 3 occlusal roughness (in c) at 1 Y	5%	25.00%
Carrillo de Albornoz et al. 2014 [67]	N/A	2 (in t)	No	No	8 %	8.00%
Baldini et al. 2016 [68]	N/A	No	No	No	No	0.00%
Paolantoni et al. 2016 [69]	N/A	2 (one piece) 1 (two-piece)	No	3 all-ceramic (one piece) fractured (26 M)	10.3%	8.11%
Wittneben et al. 2017 [58] Wittneben et al. 2020 [59]	N/A	No	No	1 crown fracture (in t) at 1 Y 1 major ceramic chipping 1 crown replacement due to esthetics at 3 Y (in c)	No	7.69%
Amorfini et al. 2018 [73]	N/A	1 in CR	2 one in each	1 chipping in SR 1 crown decementation	3%	15.63%
Eisner et al. 2018 [49]	USPHS	No	1 (in t) at 3Y	2 (one in each group) minor chipping 6 (4t/2c) occlusal wear at 3 Y	5% (same as 1Y)	45.00%
Thoma et al. 2018a [54]	USPHS	No	No	2 minor chipping in CR at B 1 poor marginal adaptation in CR at B and 6M 50% contact points alpha score at B and 6M	N/A	9.09%
Heierle et al. 2019 [53]	USPHS	1 in SR	No	1 veneer fracture in SR 4 in each group occlusal wear	3.7%	14.71%
Laass et al. 2019 [50]	USPHS	No	No	12 slight occlusal roughness (5t, 7c) 2 larger occlusal roughness 2 minor chipping	5% (same as 1Y)	80.00%
Kraus et al. 2019 [57]	USPHS	6 (2 in CR and 4 in SR)	No	Slight occlusal roughness: 4 in SR and 9 in CR Minor chipping in 1 in CR	13.6%	45.45%

*SR* screw retained restoration, *CR* cemented restoration, *t* test, *c* control, *B* baseline, *M* month, *Y* year

#### Esthetic outcomes

Eleven studies (14 articles) assessed esthetic outcomes (Table 5). The indices used for this purpose were the PES (pink esthetic score), the PES/WES (pink and white esthetic scores), the ICAI index (implant crown esthetic index), and the CIS index (Copenhagen index score).

**Table 5:** Esthetic outcomes

Study	Indices	Esthetic outcomes	
Galluci et al. 2011a [51] Galluci et al. 2011b [52]	PES, WES	PH, CLt, Cli, KMi, KMt similar between groups (p > 0.05) Significant increase in the mean PH (mesial and distal) between B and CI and between CI and 1 Y Mean CLi between CI and 1 Y p > 0.05, between 1 Y and 2 Y significant recession Mean KM p > 0.05 between groups PES, WES between groups P > 0.05. PES was higher than WES (total score approximately 13)	
Hosseini et al. 2011 [66]	CIS	Crown morphology between groups (p > 0.05) Color match better for ceramic group (p = 0.03) Mucosal dicoloration and PI p > 0.05 between groups (papilla index improvement from B to 1 Y in both groups) Overall CIS between groups p > 0.05	
Brandenberg et al. 2017 [48]	Modified PI	PI increased between B and 6 M and then slightly decreased (p > 0.05) Implants lower PI than natural teeth (p < 0.05) 1mm recession in one implant (control)	
Carrillo de Albornoz et al. 2014 [67]	ICAI PI	ICAI-crown scores: satisfactory in t group, moderate in c group (p > 0.05) ICAI-mucosa scores: improved color and surface of soft tissues with zirconia (p = 0.065) Zirconia: higher PI (p = 0.053)	
Patil et al. 2017 [62]	PES/PI	p > 0.05 between PES for t and c at B and 1 Y PES slightly improved after 1 Y in both groups (p < 0.05) p > 0.05 between PI of t and c (positive association between papillary fill and bone height between implant and tooth)	
Baldini et al. 2016 [68]	ICAI and PI	ICAI-mucosa scores: higher at 1 M (p = 0.01) for zirconia group (improved color and surface of soft tissues). Both groups: increase of PI between B and 12 M (p < 0.05)	
Bittner et al. 2020 [65]	N/A	Mean recession at 6 M (p = 0.60): Pink, thin: 1.37 mm/pink, thick: 1.28 mm Gray, thin: 1.99 mm/gray, thick: 1.13 mm The recession and collapse showed no correlation to color	
Wittneben et al. 2020 [59]	mPES and WES	1 Y: Mean PES: group A: 7/group B: 7.65 Mean WES: group A: 8.28/group B: 8.50	No difference over time
		3 Y: Mean PES: group A: 7.76/group B: 7.32 Mean WES: group A: 8.88/group B: 8.56	PES higher between B and 3 Y for group A (p = 0.04)
		Significant differences in implant crown length between B and 3 Y (p = 0.004) and 6 M and 3 Y (p = 0.012)	
Amorfini et al. 2018 [73]	Implant crown/tooth crown index PI PES/WES	Implant crown and tooth crown values similar between groups/p = 0.01 for intragroup changes in 10 Y PI increased in both groups at 2 Y (p < 0.05) and then stabilized PES 7.5 (SR), 7 (CR) (p > 0.05) WES 7.9 (SR), 7.4 (CR) (p > 0.05)	
Eisner et al. 2018 [49]	Modified PI	p > 0.05 for PI between groups at 3Y and for intragroup changes (c slight increase in PI/ t slight decrease in PI mesially) No recession in t at 3Y/ in c recession at 3Y −0.11 mm (p = 0.02)	
Thoma et al. 2018a [54]	Modified PI	Slight improvement in PI in both groups over time (p > 0.05) and no difference between groups Crown height stable over time MT slightly superior in SR group (p > 0.05)	
Laass et al. 2019 [50]	Modified PI	mPI increased over time in the c and decreased in the t	
Kraus et al. 2019 [57]	Modified PI	mPI: p > 0.31 between two groups Median mucosal level changes were 0.0 mm at both SR and CR between B and 3 Y (p > 0.44)	

*KMt* width of the buccal keratinized mucosa (gingiva) at the adjacent teeth, *CLt* distance between the mid-facial gingival margin and the incisal edge of adjacent teeth, *KMi* width of the buccal keratinized mucosa at the implant site, *Cli* distance between the mid-facial gingival margin and the incisal edge of implant crown, *PH* papilla height, *B* baseline, *Y* year, *M* months, *CR* cemented restoration, *SR* screw retained restoration, *CI* crown insertion, *t* test, *c* control

**Fig. 2:**
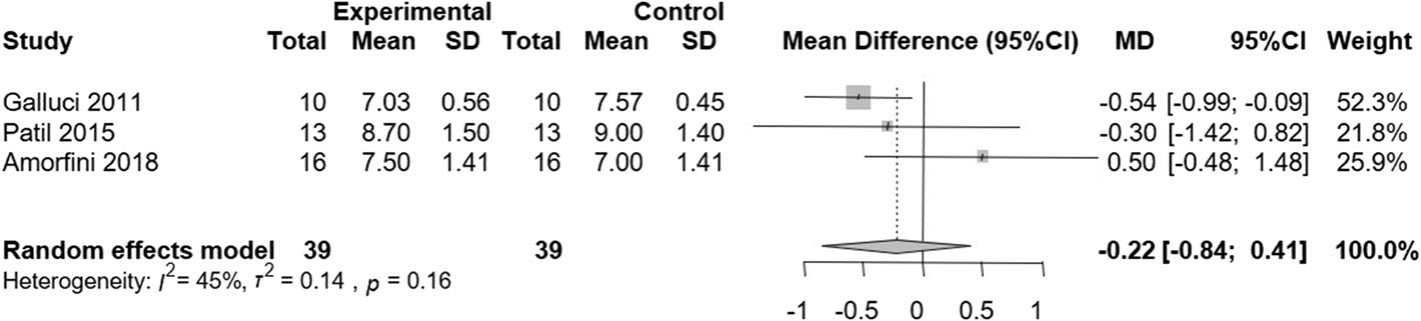
Forest plot of the overall mean difference in PES between experimental and control groups

The PES and PES/WES had good scores of a range of 9-10 and 13-18 respectively. Four studies used PES while three of them contributed to the estimation of overall mean difference in PES between experimental and control group. Wittneben et al. [59] was excluded from the quantitative synthesis since there was not sufficient data for the extraction of mean, standard deviation, and 95% confidence intervals of PES at each group. Summary measures were combined using random-effects models to minimize effects of between study heterogeneity. Heterogeneity was assessed using the I^2^ statistic and was distinguished as low (≤ 25%), moderate (25-75%), or high (≥ 75%); a p value < 0.10 was considered statistically significant. Because of the limited number of the studies, publication bias was not evaluated; Egger’s test is not applicable in a small number of studies. The difference in mean PES was statistically significant between the two groups in one study [51]. The overall mean difference in PES between the two groups was not statistically significant (−0.22, −0.84 to 0.41) (Fig. 2). The heterogeneity was low and not statistically significant (I^2^ = 45%; p = 0.16).

The PI (papilla index) was used to assess the shape and height of the mesial and distal papillae. Generally, papillae increased through time (Table 5); one study exhibited significant improvement in the papillae in the zirconia abutment group [73]. In some cases [51, 62, 66–68], patient satisfaction was assessed using a visual analog scale (VAS). The VAS scores were generally high in the assessed studies, indicating good patient acceptance.

In 9 studies [13, 22, 23, 54, 55, 65, 70–72] (Table 6), the color of the soft tissues surrounding the abutment was assessed and compared with that of the adjacent or contralateral tooth with the use of a spectrophotometer, and the ΔΕ values were calculated. They were over the clinically acceptable threshold of 3.7 [75] in all cases, and in most studies, zirconia abutments exhibited better values than other materials. One study of experimental pink veneered abutments exhibited better ΔΕ values than those of the conventional zirconia abutments [55]. In another study, an experimental fluorescent zirconia abutment was compared to a conventional white zirconia abutment with promising results, especially close to the gingival margin [72]. Some of these studies also tried to correlate the soft tissue thickness with the color difference.

**Table 6:** Mean color difference (ΔΕ) data

Study	Mean ΔΕ values	Mean mucosal thickness (mm)	Mean ΔΕ values according to soft tissue thickness	
			Thin (≤ 2 mm)	Thick (> 2 mm)
Thoma et al. 2018a [54]	CM: 5.51 ± 1.56/SR: 7.42 ± 5.05 (p > 0.05)	CM: 3.38 ± 0.99/SR:4.06 ± 1.06	N/A	N/A
Buchi et al. 2014 [23]	t: 6.3 ± 2.2/c: 4.6 ± 1.8 (p > 0.05)	t: 1.6 ± 0.4/c: 1.8 ± 0.7 (p > 0.05)	t: 6.2 ± 1.9/c: 4.2 ± 1.6 (p > 0.05)	t: 6.5 ± 4.6/c:4.9 ± 2.1 (p > 0.05)
Thoma et. al 2016b [55]	Abutment vs control tooth: P: 7.55 ± 3.02 W: 7.52 ± 2.29 Crown vs control tooth: P: 6.16 ± 2.48 W: 8.31 ± 3.33	N/A	Crown vs control tooth: P: 4.50 ± 1.93 W: 9.72 ± 3.82 (p > 0.05)	Crown vs control tooth: P: 6.88 ± 2.45 W: 8.31 ± 2.98 (p > 0.05)
Jung et al. 2008 [13]	ΔΕ implant: 7.4-7.6 (p > 0.05 between groups) ΔΕ tooth-implant overall: All-ceramic 3.4 ± 1.4 PFM 5.2 ± 2.3 (p > 0.05) ΔΕ tooth-implant grafted (p > 0.05) ΔΕ tooth-implant non-grafted (p = 0.04) (PFM higher)	All-ceramic: 3.4±0.8 PFM: 2.9±0.9 (p>0.05)	N/A	N/A
Bittner et al. 2020 [65]	ΔΕ when change from gray to pink abutment still above clinical threshold	N/A	6M: t:6.2/c:6.14	6M: t: 8.42 /c:7.96
Martinez-Rus et al. 2017 [71]	Soft tissue level: Ti: 11.56 ± 3.4 GTi: 8.96 ± 3.1 PTi: 10.68 ± 4.2 Zr Ti-base 6.06 ± 3.2 Coronal level: Ti: 10.42 ± 6.3 GTi: 9.16 ± 6.5 PTi 8.66 ± 6.1 Zr Ti-base 5.76 ± 2.9	1.63±0.64 mm	N/A Correlation between ΔΕ and thickness Ti: p = 0.024 PTi: p = 0.048	N/A
Lops et al. 2017 [70]	Gold: 11.43 ± 4.05 Ti: 13.55 ± 6.91 Zr: 11.37 ± 4.67 (p > 0.05) *all over clinical threshold ΔΕ 8.47	2.57±0.5 mm	G: 9.82 Ti: 13.86 Zr: 10.86 (p > 0.05)	N/A
Bressan et al. 2011 [22]	G: 8.9 ± 0.4 (SE) Ti: 11 ± 0.4 (SE) (p < 0.05) Zr: 8.5 ± 0.4 (SE) *over ΔΕ 3.7	N/A	G: 8.6 ± 1.4 (SE) Ti: 9.5 ± 1.4 (SE) Zr: 7.5 ± 1.4 (SE)	G: 9.1 ± 0.8 (SE) Ti: 11.9 ± 1.2 (SE) Zr: 8.9 ± 0.7 (SE) (p > 0.05)
Thoma et al. 2017 [72]	ΔΕ abutment test: 8.27 ± 4.03 ΔΕ abutment control: 8.49 ± 3.59 ΔΕ crown test: 8.32 ± 3.57 ΔΕ crown control: 7.61 ± 4.03 (p > 0.05)	N/A	ΔΕ crown c vs ΔΕ crown t: −2.08±3.82 (p < 0.05)	ΔΕ crown c vs ΔΕ crown t: 0.75 ± 2.30

*ΔE color difference, *CR* cemented restoration, *SR* screw retained restoration, *M* months, *t* test, *c* control, *Zr* zirconia, *Ti* titanium, *G* gold, *P* pink, *W* white

#### Biological outcomes

Biological outcomes were reported in 13 studies (Table 7). Most of the studies reported stability of the plaque and bleeding on probing scores over time, while some studies reported a slight increase in probing depths over time. A study comparing submucosally veneered with non-veneered zirconia abutments exhibited increased probing depths in the veneered group [50]. The mucosal thickness of the peri-implant tissues exhibited increase over time in some cases. The occurrence of a buccal fistula was reported in 2 studies [15, 66] and 2 studies reported cases of mucositis (ceramic abutments) [57, 73], while mucosal recession was reported in 4 studies [15, 49, 52, 65].

**Table 7:** Biological outcomes

Study	Implant survival rates	Biological—clinical outcomes
Andersson et al. 2001 [15]	100%	p > 0.05 for plaque and bleeding measurements between abutments at 1 Y Buccal fistula: 1 in c, recession:1 in t/2 in c
Galluci et al. 2011b [51]	100%	p < 0.05 for FMPS at 2 Y between t and c (increased scores in the all-ceramic group) FBIC p < 0.05 between B and CI at 1 Y and 2 Y
Hosseini et al. 2011 [66]	100%	p > 0.05 for mPII and mBI between groups at B and 1 Y Ceramic group 1 Y: 1 fistula, 3 suppuration on probing, 2 PPD ≥ 5 mm, 1 weak pain (most not ideal marginal adaptation—p < 0.05 for ceramic group) Metal group 1 Y: 3 suppuration and PPD ≥ 5 mm
Brandenberg et al. 2017 [48]	100%	PPD no difference between groups/ mean PPD of all implants higher at B than 1 Y (P < 0.05) BOP significantly increased in both groups after 1 Y (non-significant between group comparison) KM, MT slight increase over time (p > 0.05) At 1 Y 1 implant (control) small recession
Carrillo de Albornoz et al. 2014 [67]	100%	PPD, REC: p > 0.05 between B and 12 M or between groups FMBS: improvement for both groups from B to 1 M (p < 0.05)
Patil et al. 2017 [62]	100%	p > 0.05 for PPD between groups at B and at 1 Y
Baldini et al. 2016 [68]	100%	PPD, BOP, REC: P > 0.05 between B and 12 M or between groups MT: no changes
Paolantoni et al. 2016 [69]	100%	PI, BI p > 0.05 between groups
Thoma et al. 2018b [56]	100%	PD p > 0.05 at B, 6 M, and 1 Y between groups and intragroup changes Plaque changes non-significant BOP increase between B and 1 Y in CR white group significant BOP not significant difference between groups
Wittneben et al. 2020 [59]	100%	1Y: Low mPLI, mSBI, PD (p > 0.05 between groups) Mean KM 3.6 mm Group B KM significant difference between B and 1 Y 3Y: p > 0.05 between groups for mPLI, mSBI, PPD, and KM Within each group p < 0.05 for mPLI between time points (B and 6 M, 6 M and 1 Y, 6 M and 3 Y)
Amorfini et al. 2018 [73]	100%	2 mucositis cases (1 in each group) BOP, PPD, mPI: p > 0.05
Eisner et al. 2018 [49]	100%	PD, BOP, PI at 3 Y p > 0.05 between groups MT higher at 3Y in implants than teeth (p < 0.05) MT higher in control (p = 0.013) at 3 Y MT implant increased over time in both groups (p < 0.05) KM test 3Y 3.22 mm vs KM control 3Y 3.67 mm (p = 0.037)
Thoma et al. 2018a [54]	100%	PCR, PPD, KT stable over time and comparable between implants and teeth Median BOP at B: 33% CR, 17% SR Median BOP at 6 M: 17% CR, 17% SR
Heierle et al. 2019 [53]	100%	N/A
Laass et al. 2019 [50]	100%	p = 0.042 in PD at 5 Y between groups (increased scores in the test group)/intragroup differences between B and 5 Y not significant In the c group KM was a little wider and the MT was higher (p > 0.05) p > 0.05 for PCR, BOP
Kraus et al. 2019 [57]	81.8%	Median PD 3.0 mm in SR and 3.0 mm in CR (p = 0.664) Intragroup changes in PD from B to 3Y were statistically significant (p < 0.05) BOP and PCR: p > 0.05 between groups Median KM between implants (SR) and control teeth significant (p = 0.007) Median KM between implants (CR) and control teeth non-significant (p = 0.42) MT p > 0.05 between implants and teeth in both groups and in intragroup comparison (B vs 3 Y) One implant from CR was lost at 9 M and one at 3 Y At 3 Y 2 implants peri-implant mucositis (one in CR and one in SR)
Koutouzis et al. 2019 [74]	96.3%	Plaque, BOP, and PPD at crown placement and 1 Y (1 Y PPD: CX: 74.4% PD < 3, 25.6% PD 4-5 mm, CV: 66.7% PD < 3, 32.1% PD 4-5 mm, 1.2% PD ≥ 6 mm): no statistical difference between groups Marginal mucosa position changes p > 0.05 between groups (correlation between mucosa position and buccal bone thickness)

*PPD* pocket probing depth, *BOP* bleeding on probing, *KM* width of keratinized mucosa, *PCR* plaque control record, *KT* keratinized tissue, *GI* simplified gingival index, *mPLI or MPI* modified plaque index, *SBI* sulcus bleeding index, *REC* recession index, *FMBS* full mouth bleeding score, *DIB* distance from implant shoulder to the first bone to implant contact, *FMPS* full mouth plaque score, *FBIC* first bone to implant contact, *MT* mucosal thickness, *B* baseline, *Y* year, *CR* cemented restoration, *SR* screw-retained restoration, *c* control, *t* test

### Risk of bias within individual studies

**Fig. 3:**
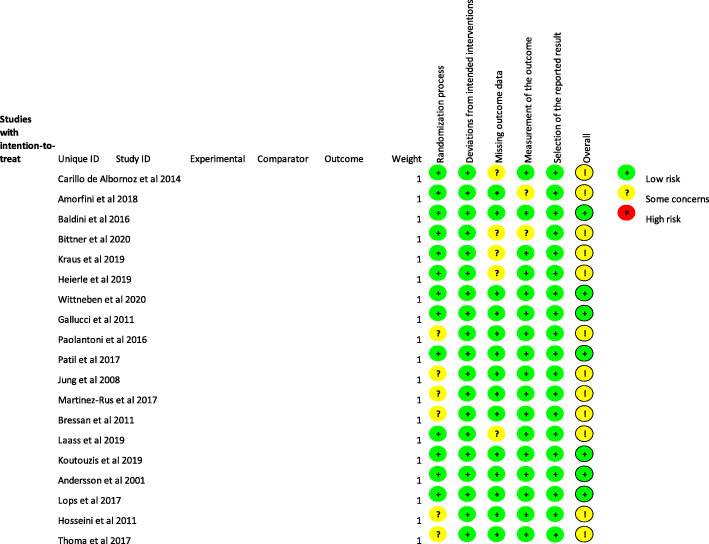
Risk of bias of the included studies

**Fig. 4:**
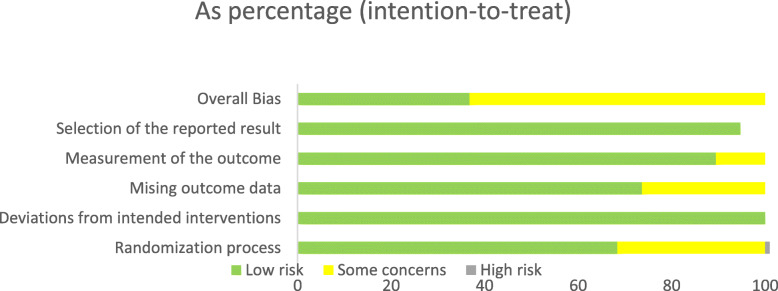
Summary of RoB2 tool results

The risk of bias of the randomized studies was assessed with the RoB 2 tool [31] and is presented in detail in Figs. 3 and 4. Seven studies exhibited an overall low risk of bias, while twelve studies raised some concerns.

## Discussion

Over the years, various implant systems, abutment, and restoration designs and materials have been introduced for the purpose of delivering a functional and natural-looking outcome for a missing tooth. Abutment’s material, method of fabrication, transmucosal macro-design, and connection, are considerations critical to the rehabilitation of a single anterior edentulous space with an implant. This systematic review, following a predetermined protocol, aimed to assess the esthetic, biological, and mechanical outcomes as well as the survival of the different types of abutments used for single-implant restorations in the anterior region. The synthesis of the individual data of the included RCTs led to the following outcomes.

### Mechanical outcomes

During the last decade, the internal connected zirconia abutments seem to be the most used abutments for anterior single implant restorations. In this review, only one study [15] reported the use of externally connected alumina abutments. Generally, the internal abutment connection is considered to reduce the incidence of screw loosening [76], which was also demonstrated in this systematic review; only 2 studies of internally connected abutments mentioned screw loosening. However, the internally connected zirconia abutment was associated with a higher incidence of fracture due to the weakest area being the neck of the abutment where the forces are concentrated. More specifically, Thoma et al. 2016 [47] reported a 5% fracture rate of the customized zirconia abutments in 1 year, which is a high percentage for such a short period of time. Similar results were noted by Heierle et al. 2019 [53] after a 3-year follow-up where one fracture of a screw-retained one-piece zirconia abutment took place. A more increased fracture rate (13.6%) was noted in a study comparing screw retained and cement retained single-implant restorations [57]. Most fractures were observed in the screw retained group, raising the question of whether the type of crown retention might influence the fracture resistance of the reconstruction. The authors proposed that the cementation gap in cemented restorations might compensate the stresses and reduce the fracture risk [57]. However, most fractured abutments occurred in premolar teeth, so the implant site might be another factor that can contribute to the increased fracture rate. It is noteworthy that in 2 studies, the ceramic abutment fracture took place during the screw tightening [15, 67]. This might be a result of stress during laboratory preparation in order to individualize the prefabricated abutments, often resulting in very thin abutment walls that might not withstand the torquing forces.

Two studies examining the same type of abutments, Baldini et al. [68] resulted in no technical problems after 1 year while Carrillo de Albornoz et al. [67] reported zirconia abutment fractures after the same time period. Both zirconia and titanium abutments were restored with metal-ceramic crowns in the first study [68], while all-ceramic crowns covered the zirconia abutments in the other study [67]. The type of restoration may have been a factor on the stress distribution on the abutment. However, this is only a speculation and the short follow-up period (1 year) further reduces the strength of the findings. It must be noted that no fractures were reported in the studies with internally connected titanium abutments, indicating the higher strength of these abutments [77, 78].

Implant diameter has been postulated to influence the fracture resistance of an abutment, with narrow implants being more susceptible [77]. In this systematic review, out of 7 narrow abutments, 2 fractures were reported [67], while one study of regular diameter implants reported a 3% fracture rate [73]. In the other relevant studies, the implant diameter was not specified, therefore making the investigation of this hypothesis difficult due to lack of adequate evidence.

Τwo studies reported a new type of hybrid abutment, a zirconia abutment cemented on a titanium base [71, 72]. This type of abutment is considered to improve the performance of zirconia abutments by mechanically reinforcing the abutment and providing a more stable internal connection. No mechanical outcomes were reported in the included studies. Regarding the zirconia abutments that were subgingivally modified with pink ceramic to improve esthetics, only one abutment fractured [49], although an in vitro study [79] concluded that veneering the zirconia could influence the flexural strength of the abutment.

Due to the heterogeneity of the studies included, the different follow-up periods, and the fact that some studies did not report the time of abutment failure, it was difficult to calculate the abutment survival rate. However, the percentage of the abutment fracture requiring replacement was considered the most significant complication and was estimated at 4.26%.

### Esthetic outcomes

The effect of the abutment and restoration on the final shade of the peri-implant mucosa has been explored in some included studies. The most objective measurement of this effect is the spectrophotometric measurement and the calculation of the color difference (ΔΕ) of the implant mucosa or the implant crown to that of the natural tooth. Four studies [13, 22, 70, 71] compared the esthetics of titanium, gold, and ceramic (zirconia or alumina) abutments. All abutments exhibited clinically visible tissue discoloration with titanium abutments exhibiting significantly higher ΔΕ than gold or zirconia.

The tissue thickness has been considered to influence the effect of the abutment on the final shade of the peri-implant mucosa [12], although it was not fully supported by the included studies. Martinez et al. [71] compared titanium, gold-hued titanium, pink-hued titanium, and zirconia with Ti-base abutments and interestingly, the gold titanium esthetics were almost comparable with zirconia, especially in thicker biotypes, making it a better choice than unhued titanium and even pink titanium. In this study, it was noted that the zirconia abutments were cemented on a Τi-base that might result in some shine through and influence the results, reducing the color difference between gold and zirconia. Another study compared pink and gray titanium abutments placed on implants with either pink or gray-hued collar [65]. The pink abutments reduced the a* value ΔΕ, resulting in redder gingiva that appeared more natural, regardless of the implant color, especially in thin biotype cases.

It is therefore evident that zirconia abutments are more esthetic. However, due to the bright white color of these abutments, they cause discoloration in the soft tissues, especially if compared to the mucosa of the natural teeth. For better esthetic integration, techniques modifying this color of zirconia by either veneering it with colored ceramic or by using fluorescent zirconia were proposed. The pink veneered zirconia abutments were compared with the conventional white ones and a non-statistically significant, more intense discoloration was noted with the veneering, possibly as a result of the translucency of the pink ceramic, which reduced the L* value and resulted in a grayish discoloration [23]. A subsequent study at the same university used a slightly more opacious pink ceramic with marked improvement [55]. The veneering technique improved esthetics regardless of the tissue thickness, although the ΔΕ was still above the clinically acceptable. It was confirmed that the tissue thickness influences the color integration of the abutment, and the non-veneered abutments exhibited better color match with the natural teeth in cases of thickness > 2 mm. It was also noted that the lowest color difference was recorded in thin biotypes with pink veneering, supporting the hypothesis that the modification is especially important in cases with thin tissues. Finally, no significant difference was found between fluorescent zirconia abutments compared with conventional one-piece zirconia abutments [72]. However, in thin biotypes, white zirconia showed better results, maybe due to the fact that the fluorescent abutments were cemented on a titanium base, which may have been visible through the thin tissues, decreasing the lightness. The use of two different abutment designs (two-piece with Ti-base and one-piece) was a limitation of this study, not allowing to fully investigate the impact of the fluorescence.

Another study [58] investigated the influence of the crown retention (screw-retained vs cement retained) on the esthetic performance of zirconia abutments; however, no significant difference was noted between the two groups and all abutments resulted in a clinically visible discoloration of the mucosa, even though the mean mucosal thickness was over 3 mm.

The esthetic outcomes of the different implant abutments were also assessed using relevant indices (PES, WES, ICAI, CIS) as well as recording patient satisfaction. Most studies comparing the esthetic outcome between ceramic and titanium abutments revealed no significant differences [51, 67, 68]. On the contrary, Hosseini et al. [66] observed a better crown color match for all-ceramic restorations, although the mucosal discoloration was evident with both materials.

Αn increase in the papilla index score was observed over time [51, 67, 68]. The papillary response was probably influenced more by the abutment shape than the material as no significant differences in PI scores were noted between the ceramic and titanium abutments. The comparison between the types of retention (screw versus cement retained) of ceramic abutments [54, 57, 73] or between CAD/CAM and prefabricated abutments [58] exhibited similar good esthetic results. Nevertheless, in the clinical experience of the author’s customized abutments provide better support to the soft tissues, which is especially needed in high scalloped tissue cases. Additionally, two studies of modified zirconia abutments [50, 57], revealed no significant difference between the tested abutments and the conventional ones. Another study [74] comparing the effect of macro-morphology (convex versus concave) of titanium abutments on esthetics resulted in similar results on both groups and an increase in papilla was noted over time.

With respect to patient satisfaction, in most cases, the scores were quite high and no difference between materials was noted. In studies where the patient satisfaction was compared with the clinician’s assessment, a discrepancy was noted; the patients were more satisfied than the dental practitioners, confirming the notion that individuals perceive esthetic deviations to a lesser extent [66, 67]. However, when patients were dissatisfied, crown color and tissue morphology were the main reasons they reported [66].

### Biological outcomes

The findings of this systematic review led to similar scores of probing depths, bleeding on probing and mucosal stability between ceramic and titanium abutments [15, 67, 68] which is comparable with the findings of a previous systematic review [27]. However, Hosseini et al. [66] concluded that the most biological complications were related to all-ceramic restorations, most likely because of the lack of ideal marginal adaptation, due to the use of pre-sintered zirconia copings.

The impact of the abutment macro-morphology on the soft tissue stability and health was also assessed. No significant difference in mucosal alterations was present between curved or concave and convex titanium abutments [60, 74]. Therefore, the new concept of manufacturing an abutment with a concave transgingival morphology in order to improve the soft tissue stability was not supported by this review.

The effect of the type of retention on the biological integration of the implant abutment was also explored. The cemented restorations have been associated with biological complications due to the marginal fit of the crown and the excess cement management [80]. Kraus et al. [57] found no statistically significant difference between the two restorative types after 3 years; the cement retained restorations were associated with two catastrophic failures though. The statistical significance of the findings could have been influenced by the small sample size and the high drop-out rate. On the contrary Amorfini et al. [73] found that the frequency of biological complications for zirconia abutments was similar for both retention types. Probably, the equigingival delineation of the margin of the CAD/CAM abutments and hence the easy removal of cement excess, prevented the development of biological complications.

The influence of abutment customization on the biological outcomes was assessed by Wittneben et al. [59] and no significant difference was noted. Still, it has been argued that in cases where a cement retained restoration is considered, the abutment customization seems to be preferable as it enables the margin be placed at the appropriate position.

The modern concept of submucosally veneered zirconia abutments was also investigated in this review, although only one clinical trial [50] was available from which to extract the data. After 5 years, the probing depths around the veneered abutments were significantly higher than around the conventional ones. The veneering of zirconia has been associated with surface roughness increase [81], favoring plaque accumulation and subsequent periodontal inflammation. Notably, although the probing depths were increased, the marginal bone levels were not influenced. More randomized trials with larger sample sizes and a longer follow-up period are needed to assess the association of the veneering technique with possible biological effects.

Notably, in a recent systematic review [82] has shown that the different types of implant-abutment interface also affect the mechanical and biological outcome of single-tooth implants in the esthetic area. More specifically the Morse Taper design performed better for marginal bone loss, success, and survival while internal hexagon had the best PES/WES score. This study [82] as well as the current systematic review highlights the need for more robust results from well-designed and executed randomized controlled clinical studies.

## Conclusions

The following conclusions can be drawn from this systematic review:

- Abutment failure, due to fracture, was associated with ceramic abutments and a mean rate was calculated at 4.26%.
- Fracture of the ceramic abutment was more common in the neck area of the internally connected one-piece abutments or in the weakened area due to over-preparation of prefabricated abutments.
- The assumption that ceramic abutments placed on narrow implants are more prone to fractures, was not supported.
- The most common mechanical complication was abutment screw loosening; however, this was considered a minor complication.
- Titanium abutments caused significantly more discoloration to the soft tissues than ceramic abutments, while hueing (gold or pink) slightly improved their color performance.
- Gold or gold hued abutments performed almost like zirconia abutments.
- Limited correlation was noted between soft tissue thickness and color difference.
- Submucosal veneering sometimes seemed to improve the color match of zirconia abutments; however, the translucency and color of the veneering material seemed to influence the outcome. Nevertheless, more randomized trials are needed to assess the esthetic value of this modification and to assess its biological impact, which at this point raised some concerns.
- Similar biological complications were noted for metallic and ceramic materials.
- The type of restoration retention did not appear to significantly affect the biological outcome; however, as screw retained restorations provide more retrievability, they might be a better choice when possible.

## Data Availability

Not applicable

## Abbreviations

CAD/CAM: Computer-aided design and computer-aided manufacturing
PRISMA: Preferred Reporting Items for Systematic Reviews and Meta-Analyses
PROSPERO: International Prospective Register of Systematic Reviews
PICO: Participants/intervention/comparison/outcomes tool
RCTs: Randomized controlled trials
CENTRAL: Cochrane Central Register of Controlled Trials
MeSh: Medical Subject Headings
ROB 2: Cochrane Risk of Bias tool
No_ patients: Number of patients
No_ abutments: Number of abutments
Zr: Zirconia
Ti: Titanium
PFM: Porcelain fused to metal
N/A: Not applicable or not available
USPHS: United States Public Health Service criteria
SR: Screw retained restoration
CR: Cemented restoration
t: Test
c: Control
B: Baseline
M: Month
Y: Year
PES: Pink esthetic score
WES: White esthetic score
ICAI: Implant crown esthetic index
CIS: Copenhagen index score
PI: Papilla index
VAS: Visual analog scale
KMt: Width of the buccal keratinized mucosa (gingiva) at the adjacent teeth
CLt: Distance between the mid-facial gingival margin and the incisal edge of adjacent teeth
KMi: Width of the buccal keratinized mucosa at the implant site
Cli: Distance between the mid-facial gingival margin and the incisal edge of implant crown
PH: Papilla height
CI: Crown insertion
ΔE: Color difference
G: Gold
P: Pink
W: White
